# Anæsthesia in Dental Practice

**Published:** 1870-12

**Authors:** 


### Editorial.

### ANÆSTHESIA IN DENTAL PRACTICE.

THE useful is ever liable to abuse. This is as true of anæsthesia as of other things. For two reasons it is more likely to be abused by dentists than by physicians; they perform painful operations oftener than physicians do; and, though but little varied, for the time they last, they are among the most painful known to surgery. It is not strange, then, that anæsthesia was discovered by a dentist, nor is it strange that dentists resort to it much more frequently than physicians do; and it is fortunate for humanity that the more dangerous chloroform has been mainly superseded in dental surgery by the less dangerous protoxyd of nitrogen.

This gas, commonly called nitrous oxyd, has been a plaything for three-quarters of a century, and tame lions have been used for the same purpose much longer, the use of either, in this way, proving that the fools are not all dead. The fact of nitrous oxyd having been so long thus used illegitimately, and its having acquired the name, "laughing gas," encourage the ignorant to administer it, and the fools to take it. All this is very unfortunate. Many try to make the gas who have no correct ideas on the subject; many more administer what they call nitrous oxyd, who have no true notions of the nature of anæsthesia, or of what they use. The consequence is that often the lining membrane of the air cells is cauterized, or worse, the red globules share the same fate, and the function of respiration is, for hours or days, almost destroyed. The shock to the brain and nervous system, in such cases, is fearful; but as the patient usually gets away from the office before the worst symptoms manifest themselves, the dentist usually escapes censure, and the physician generally ascribes the morbid symptoms to something else. The constitutions injured in this way, that is, by the use of impure gas, or the improper use of pure, are almost countless. It is a matter of surprise that physicians give so little attention to this; but, as a general rule, they appear to be equally ignorant of the therapeutic properties of nitrous oxyd.

Another bad effect has followed the use of nitrous oxyd. Regarding it as safe, and not unpleasant, hundreds of silly people have been persuaded to sacrifice comparatively good natural teeth, that their places might be occupied by artificial substitutes. The quacks reap a harvest; and some who oppose them indulge in such extreme statements, that their influence all goes on the side of the quack. For example, the man who states that the extraction of a tooth, under any circumstances, is a crime, states what he knows is not true; and the quack and his victim both know that he is not aiming to tell the truth, and are, accordingly encouraged to go on with their murderous mutilation.

When properly prepared, and properly administered, no other anæsthetic, and indeed no other article of the Materia Medica, responds more promptly and uniformly to reasonable expectations; but before its advantages can be fully realized, both the profession and the public must be much better educated in regard to it. The public must learn that the use of it is not "the best joke of the season," and that it is not to be trifled with. The profession must learn that the preparation of it requires some little chemical knowledge, and its administration a good deal of common sense. They must learn that delirium and unconsciousness are not parts of, nor essential to anæsthesia. They must learn that nitrous oxyd has no tendency to darken the red corpuscles, and that, therefore, if the complexion is darkened, they are administering impure gas, or smothering the patient. They must learn to operate as *carefully* and deliberately as if the patient were in his natural state. They must learn to regard as *failures* many of their cases that they now report as "perfectly successful." They must know vastly more about the function of respiration than they now do, so that they will realize the deadly nature of nitric oxyd, chlorine, and carbonic acid, when inhaled. They must learn that the old plan of administering to the patient his own breath with the gas, is both filthy and deadly, and that all that has been written, with such administration as a basis of observation, is valueless as authority. When all this is learned, nitrous oxyd will be a blessing. As generally used at present, it is a curse. W.

